# SARS-CoV-2 infection and human semen: possible modes of contamination and transmission

**DOI:** 10.1186/s43043-021-00063-6

**Published:** 2021-06-21

**Authors:** Koushik Bhattacharya, Lipika Das Mukhopadhyay, Ratnadeep Goswami, Sulagna Dutta, Pallav Sengupta, Tulay Irez, Habibah Abdul Hamid, Alak Kumar Syamal

**Affiliations:** 1 Department of Physiology, Rungta College of Dental Sciences, Bhilai, Chhattisgarh India; 2 Department of Obstetrics and Gynecology, KPC Medical College, Jadavpur, Kolkata, West Bengal India; 3 Department of Obstetrics and Gynecology, Matrisadan Hospital, & ILS Hospital, Dumdum, Kolkata, West Bengal India; 4 Department of Oral Biology and Biomedical Sciences, Faculty of Dentistry, MAHSA University, Jenjarom, Selangor Malaysia; 5 Department of Physiology, Faculty of Medicine, Bioscience and Nursing, MAHSA University, Jenjarom, Selangor Malaysia; 6 Department of Histology and Embryology, Medical Faculty, Istanbul Yeni Yuzyil University, Istanbul, Turkey; 7 Department of Obstetrics and Gynecology, Faculty of Medicine and Health Sciences, Universiti Putra Malaysia, Serdang, Selangor Malaysia; 8 Post Graduate Department of Physiology, Hooghly Mohsin College, University of Burdwan, Bardhaman, West Bengal India

**Keywords:** COVID-19, Male infertility, SARS-CoV-2, Semen infection, Sexual transmission

## Abstract

**Background:**

Coronavirus disease 2019 (COVID-19), caused by the severe acute respiratory syndrome-coronavirus-2 (SARS-CoV-2), has turned into a global pandemic with multitudinous health impacts.

**Main body:**

In light of the higher vulnerability of men to COVID-19 than women, there is rising concerns on the impact of SARS-CoV-2 infection on male fertility and possibilities of seminal contamination and transmission. The pandemic has attributed to the brief suspension of many fertility clinics and pathology laboratories, though many remained functional. Few reports reflect that SARS-CoV-2 can contaminate the semen of COVID-19 patients as well as that of recovering patients. The viral invasion into the testis may be due to the disrupted anatomical barriers of the testis by the inflammatory responses, and the persistence of the virus in the semen may be facilitated by the testicular immune privilege. Since SARS-CoV-2 is an enveloped RNA virus, it is also theoretically possible that this virus can remain viable in the semen samples even after cryopreservation with liquid nitrogen.

**Conclusion:**

The present review emphasizes the possibilities of seminal dissemination of SARS-CoV-2 and thereby the chances of its sexual transmission. These perceptions and predictions are to facilitate immediate necessary actions to improvise the standard precautionary procedures for laboratory practices, including semen analysis or processing the semen sample for fertility treatments.

## Background

The pandemic of coronavirus disease 2019 (COVID-19) by the severe acute respiratory syndrome-coronavirus-2 (SARS-CoV-2) has taken the world to the edge of health emergency. This β-coronavirus was first identified in December 2019 in Wuhan city, China [1]. On 11th March 2020, COVID-19 was declared a pandemic by the World Health Organization [1]. SARS-CoV-2 belongs to the family of Coronaviridae, along with SARS-CoV and Middle East Respiratory Syndrome-Coronavirus (MERS-CoV). These viruses have been reported to cause similar morbific impacts on the lower respiratory system. Transmittable SARS-CoV had been detected in blood, urine, and stools while MERS-CoV had been found only in respiratory fluids [2]. Inexplicably, COVID-19 occurrence differs with gender, as men have been seen to be more susceptible to it than women [3]. In the light of higher vulnerability of men to COVID-19, there are rising concerns on the impact of SARS-CoV-2 infection on male fertility as well as possibilities of seminal contamination and transmission.

Fertility treatments have been put on ice by many countries to alleviate the risk of spreading the infection, following the guidelines by the prominent international professional bodies (e.g., the American Society of Reproductive Medicine and the European Society of Human Reproductive and Embryology). The virulence of SARS-CoV-2 along with the ignorance of its impacts on assisted reproduction, deferral was the safest course of action.

## SARS-CoV-2 in testis and possibilities of seminal contamination

SARS-CoV-2 shares 79% similarity in genetic ancestry with the SARS-CoV, and both these viruses recognize the same cellular entry receptor, angiotensin converting enzyme receptor-2 (ACE2) [4, 5]. The transmembrane protease, serine-2 (TMPRSS2) mediates most of the priming of viral spike proteins with ACE2 [6]. The testes reportedly are among the tissues with the highest ACE2 mRNA and protein expressions, which brought along a possibility of potential threat to male fertility by SARS-CoV-2 infection [6]. Reports depict significantly higher expressions of ACE2 expression in the testes compared to that in the ovaries [7], which explain higher chances of the infection in the testes [8]. It is also evident that activation of TMPRSS2 gene is dependent upon androgen receptor. Moreover, for both androgen receptor and ACE2, gene loci are localized in chromosome-X. Genetic polymorphisms of this chromosome and subsequent endogenous androgen actions reportedly are associated with TMPRSS2 gene transcription, ACE2 activation, and thus facilitation of viral invasion in target cells [9]. SARS-CoV-2 invasion in testicular cells is not yet evidenced, with elusive concept on the testicular viral dynamics and an in situ hybridization reporting non-recognition of viral genomic materials by testicular cells [10]. However, immunohistochemistry showed IgG deposition in testicular cells [10], which is an evidence towards secondary immune reaction mediated testicular effects of SARS-CoV-2 [11]. The testicular effects of SARS-CoV-2 may be impaired Leydig cell, Sertoli cell, and sperm functions, possibly via oxidant-sensitive inflammatory pathways rather than via influence upon the endocrine axes [11–13] Although data supporting SARS-CoV-2 invasion in testicular cells are elusive, Zhao et al. had reported the presence of SARS-CoV in the Leydig cells and testicular epithelial cells [14].

The COVID-19 outbreak has been very sudden and novel in nature, and despite a great surge in research on SARS-CoV-2, information on most of its aspects remain elusive. Thus far, there are mixed findings regarding the presence of SARS-CoV-2 in semen. In 2005, the European Union issued guidelines for the mandatory screening of all the patients for three blood-borne viruses, namely the hepatitis B and C (HBV, HCV) and the human immunodeficiency virus (HIV), prior to embarking on any form of assisted reproductive techniques (ART). Recently, mosquito-borne viruses have also shown its effects on fertility [15]. For instance, an asymptomatic man for up to 1 year of his post recovery period has been detected with the Zika virus (ZIKV) RNA in the semen [16, 17]. Especially, viruses with high titers in blood (viremia) have a great probability of being shed into other body fluids such as sweat, urine, feces, breast milk, and semen. Around 27 viruses have been detected in human semen till date. Hence, there could be chances of SARS-COV-2 also shedding into semen and potentially remaining infective and precarious. Also, the immune responses in male reproductive tract, an inflammatory propagation that may alter the blood-testis barrier and the structural stability of the virus along with the viremia, contribute in the existence of viruses in semen [18]. However, it may not be requisite that the presence of viruses in semen is a function of cognate receptors for the infection within the testis or even the capability of the virus to replicate in the male reproductive system. Finally, considering the paucity of data available on SARS-CoV-2, it is circumspect to assimilate information from the viruses belonging to the same family.

The blood and feces samples in COVID-19 patients has shown the presence of the virus [5, 19]. It is notable that in fecal specimen very low titers of SARS-CoV-2 have been detected by RT-PCR with high cycle threshold/Cq values ranging from 36–38 [19]. Considering this, there is very minute risk of substantial shedding of this virus in the human semen. However, the spermatogonia, Leydig cells, and the Sertoli cells [6] have a dominating expression of ACE-2 bringing the reproductive organs into the target radius of SARS-CoV-2 infectivity. A study has reported the presence of viral RNA in the semen or the testicular tissue in one out of 10 deceased patients [20], and another including 34 patients with mild symptoms [21]. Though in these studies, small sample size and selection bias, including those with severe viremia, may have influenced the given results. But, another cohort study has reported the presence of SARS-CoV-2 in four out of 15 chronically ill and in two out of 23 recovering men [22], which reflect that SARS-CoV-2 can contaminate semen of COVID-19 patients as well as that of recovering patients (Table 1 [20–32];). The viral invasion into the testis may be due to the disrupted anatomical barriers of the testis by the inflammatory responses, and the persistence of the virus in the semen may be facilitated by the testicular immune privilege. However, several studies till date have denied the viral presence in the semen of men in different phases of recovery [20, 21, 23, 28–30, 32]. In brief, due to the lack of strong valid information from large-scale cohort studies, seminal presence of SARS-CoV-2 in the severely infected patients cannot be confirmed. However, the few abovementioned reports on seminal presence of the virus definitely raise concern regarding the sexual transmission of the virus. For further verification of this premise, detailed studies are required. As of now, there is no valid evidence of the virus surviving beyond several months after the infection, which was not the case with ZIKV [16, 17]. In a case study of six men who died of SARS-CoV infection, the number of spermatozoa severely was seen to decline along with the destruction of germ cells. Testicular autopsy showed orchitis in all the six patients, along with damaged blood-testis barrier and direct destruction of seminiferous epithelium [10]. There is also a possibility of hyperthermia due to fever, secondary infection, hypoxia, and steroids being the key mediators of testicular damage in SARS-CoV-2 patients [33]. Preliminary studies had also shown that COVID-19 could impact on the male reproductive system failure [34].

**Table 1:** Studies reporting the relation between COVID-19 and possible infectivity through semen and sexual transmission

Authors	Study design	Subjects	Findings
Pan et al. [21]	Observational, cross-sectional study	34 (adult male)	1. SARS–CoV-2 was not detected in the semen of enlisted patients recovering even a month after COVID-19 diagnosis. 2. ACE-2 receptor-mediated SARS-CoV-2 entry into target host cells is unlikely to prevail within the human testis.
Nora et al. [23]	Pilot cohort study	34 (adult male)	1. Function of testis and epididymis was not likely affected by a mild COVID-19 infection, whereas semen parameter analysis seems to be impaired after a moderate infection. 2. Detection of SARS-CoV-2 RNA showed negative in semen of recovered individuals and acute COVID-19 positive patients, suggesting no viral transmission during sexual behavior as well as when performing the assisted reproductive techniques (ART).
Li et al. [22]	Cohort study	Out of total 50 patients, 38 patients were enrolled for semen testing (men of 15 years and above)	Six patients (15.8%) of total 38 resulted positive for SARS-CoV-2 from semen samples. But no significant differences were observed between negative and positive test results in respect to age, disease history of urinary or genital tract, days since onset, days since hospitalization, or days since clinical recovery.
Song et al. [20]	Descriptive study	Total 13 patients (including one patient who died in COVID-19 infection) (12 patients with age group 22 to 38 years. One patient with age 67 who died in COVID-19 infection)	All of the patients tested negative for SARS-CoV-2 RNA in collected semen samples as well as testicular biopsy (for the dead patient) concluding the absence of sexual transmission property of SARS-CoV-2 from male.
Segars et al. [24]	Systematic review and meta-analysis	79 articles were included out of 663 articles.	Reports of this article indicated the reduced sperm concentration and motility for 72–90 days following SARS-CoV-2 infection.
Paoli et al. [25]	Case study	One (31 years old)	Semen and urine samples appeared as negative for the presence of SARS-CoV-2 RNA.
Ma et al. [26]	Case control study	81 reproductive-aged men with SARS-CoV-2 infection and 100 age-matched healthy men	This study explained the direct evidence for the severity of SARS-CoV-2 infection on male sex hormones, alerting more curiosity to gonadal function evaluation among those patients recovered from SARS-CoV-2 infection, especially the reproductive-aged men.
Li et al. [27]	Case-controlled study	Autopsied testicular and epididymal specimens of deceased (*n*=6) and recovering (*n*=23) COVID-19 male patients with an equal number of age-matched controls.	Among the COVID-19 patients, a spermatogenic dysfunction was observed, which could be due to a sequel of elevated immune response in testis. Besides autoimmune orchitis, which was recorded in a couple of COVID-19 patients.
Kayaaslan et al. [28]	Cohort study	Patients with acute-stage of COVID-19 infection (n =16)	SARS-CoV-2 RNA was not detected in semen. Decreased serum FSH, LH, and testosterone levels have been found in COVID-19 group compared to controls; significant reduced sperm morphology in COVID-19 group compared to controls; no significant differences between groups after treatment.
Guo et al. [29]	Cohort study	COVID-19 patients in acute and recovery phase (n=23)	SARS-CoV-2 RNA was not detected in semen
Temiz et al. [30]	Case-controlled study	Control (n=10), COVID-19 pre-treatment (n=10) and post-treatment (n=10)	No SARS-CoV-2 RNA detected in semen.
Gacci et al. [31]	Cohort study	Sexually active men recovered from COVID-19 (n=43)	SARS-CoV-2 RNA detected in semen of one patient. 25% found to be oligo-crypto-azoospermic that was related to COVID-19 severity; 76% found to have increased seminal IL-8.
Ruan et al. [32]	Case-controlled study	Patients recovering from COVID-19 (n=74). Age-matched healthy controls (n=174)	No SARS-CoV-2 RNA detected in semen or urine. Significantly reduced sperm concentration, total sperm count, and total motility compared controls.

Recently, a single-center observational study by Li et al. was conducted on recovering COVID-19 male patients as well as using testicular and epididymal tissues of deceased COVID-19 patients [27]. The study revealed testicular congestion, interstitial edema, exudation of red blood cells, and T-lymphocyte (CD3+) and macrophage infiltration in the testes of the deceased COVID-19 patients. These corroborations point towards increased inflammatory responses in the testis (orchitis) along with epididymides (epididymitis) [27]. The same study traced ACE2 protein expression in the testicular cells of men who succumbed to COVID-19 and reported high ACE2 expressions in the Leydig cells [27]. ACE2 mRNA expression has also been shown to be high in spermatogonia [6, 21]. All these evidences along with a prominent rise in apoptotic cells within seminiferous tubules in deceased COVID-19 patients owing to the substantial germ cell destruction suggest SARS-CoV-2-mediated impairment of spermatogenesis. For analysis of the effect of COVID-19 on testicular functions in the recovering patients, semen samples were collected from twenty-three COVID-19 inpatients [27]. A week prior to the semen collection, all the patients were tested positive for SARS-CoV-2 RNA in the throat swab. The semen samples tested negative for SARS-CoV-2 RNA, but semen analysis showed that nine out of 23 COVID-19 inpatients (39.1%) had sperm concentration below 15× 10^6^/ml, which corresponded to the recommended standards of WHO for diagnosing oligozoospermia, and 60.9% had significantly higher number seminal leucocytes [27].

However, in situ hybridization gave no absolute evidence of the viral RNA in the testis. Xu and colleagues have reported that the elevated levels of lymphocytes and macrophages in the testicular intercellular tissue might be due to secondary inflammatory responses to SARS-CoV-2 [10]. When 34 males recovering from mild symptoms were examined, 20% of them reported scrotal discomfort implicative of orchitis but studies remained inconclusive [21]. In order to preserve fertility of the COVID-19-affected men, while taking into account their holistic well-being, health care professionals should probe and investigate for the mildest of symptoms and the slightest possibility of presence of similar disease sequelae.

Evidences pertaining to the immune-protection by the intact blood-testis barrier have ruled out the possibility of SARS-CoV-2 in semen of mildly affected men [21]. The actual risk lies with the patients who are severely affected due to exaggerated immune responses compromising the testicular immune privilege. However, studies on men at advanced stage of COVID-19 is not available owing to difficulties in obtaining semen samples from those men as it is very less likely for them to ejaculate. Furthermore, the ethical and practical challenges one may face while collection of the semen sample shackle the pursuit of further studies for verifiable evidence of the presence of this virus and its colligative consequences in the male reproductive tract.

## SARS-CoV-2 transmission during semen analysis

Due to the prominence of the SARS-CoV-2 pandemic, many fertility clinics and pathology labs have undergone a brief suspension, though many other labs still remained functional. This resulted in the closure of many diagnostic semen analysis processes, yet many pathology labs continued to perform post-vasectomy semen analysis, as they are considered to cause minimum risk of transmission [35].

As discussed earlier, a couple of preliminary studies show the possibility of transmission of coronaviruses through semen [10, 22]. Thus, it is mandatory to take certain precautions during laboratory practices while performing any type of semen analysis or semen processing for fertility treatments. For this purpose, it is necessary to add new techniques to standard procedures. Perhaps it may be appropriate to apply triple gradient systems used in semen procedures, just like in removing HIV virus [36]. Taking into consideration the fact that SARS-CoV-2 is an airborne virus, henceforth, it is advisable for one to take proper care so as to avoid exposures to aerosols. While performing certain aerosol-generating events and using specimen containers used for the collection of semen and the tubes used for the sample centrifugation, one should take careful measures to avoid any sort of contamination of the sample from the external environment. It is also advised to keep fully compact and tightly closed lids of all the tubes and to use aerosol-tight caps of centrifuge bucket during centrifugation. Also, staffs working inside the laboratories of fertility clinics must wear appropriate personal protective equipment. Above discussed precautions must become the part of standard practice, if not already being into consideration.

## SARS-CoV-2 and cryopreservation

While observing the forms of standard practices of cryopreserving viruses in the laboratories, it has been noticed that even at ultra-low temperatures most of the viruses remain viable if stored dried, or in appropriate protein concentrations, and also in pH ranging between 7 and 8 [37]. For example, the influenza virus (an enveloped RNA virus belonging to the orthomyxoviridae family) causes similar respiratory tract pathogenesis like SARS-CoV-2 and reportedly can survive even when stored in the vapor phase of liquid nitrogen for up to 40 years [38]. Although there is scanty empirical data, it is theoretically possible that this virus can also remain viable in semen sample even after cryopreservation with liquid nitrogen, as it is an enveloped RNA virus. Till date, there is no evidence in the field of ART regarding cross-contamination of one virus to the other in contrast with storing them in liquid nitrogen or vapor phase. However, ART cryobanks are ought to maintain careful measures while storing all the samples in hermetically sealed highly secured devices, with quarantined samples segregated in cryovessels with the help of complete screening procedures, while also stipulating regulations for the associated risks in transporting the cryopreserved samples [39].

## Possibility of SARS-CoV-2 being sexually transmitted

Though SARS-CoV-2 has been detected in the semen of people who have or are recovering from the virus [40], there is currently no evidence that the SARS-CoV-2 virus is transmitted through semen or vaginal fluids. In a systematic review, Tur-Kaspa et al. have reported that SARS-CoV-2 infection is not a sexually transmitted disease (STD) [22, 41]. But, according to the information from MayoClinic, the Centers for Disease Control and Prevention has recommended to resume your normal activities (including sexual contact) following complete vaccination. They have also recommended to avoid sexual contact with anybody who does not live in the same family if the persons are not fully vaccinated [42]. However, as discussed earlier, there was evidence of SARS-CoV-2 infection with testis [40] that may attribute to disruption of blood-testis barrier leading to subsequent infection and inflammation [10, 43]. Given the presence of SARS-CoV-2 in several biological fluids, most prominently in the respiratory droplets, mucus, saliva, and feces, and the transmissibility of the virus within approximately 6 ft distance, physical sexual intimacy presents a high-risk scenario for viral transmission, particularly for non-monogamous partners who do not live with one another. Thus, further research is needed to determine if the COVID-19 virus could be transmitted sexually.

## Conclusion

Most of the reports on SAR-CoV-2 infection in men recovering in different phases of COVID-19 have denied the presence of the virus in the seminal plasma. Moreover, the studies pertaining to SARS-CoV-2 and semen infectivity mostly have small volume of sample size and no long-term follow-up. Therefore, further studies are required to investigate the detailed information about SARS-CoV-2 infectivity, pathogenesis, and its transmission to comprehend the implication in both clinical and epidemiology. Since there is possibility of semen contamination and impaired fertility parameters via SARS-CoV-2 infection, it is still advised as a major precaution for men who are recovering from COVID-19, to undergo a semen analysis test to confirm whether their fertility status has been affected by the illness or not. Moreover, the pandemic-enforced suspension of fertility laboratories may impact certain cohorts of population suffering from subfertility who may develop irreversible infertility and are therefore in emergency need to preserve sperms or undergo assisted reproduction. Thus, out of all the uncertainties, this global pandemic turns out to be a testing time specifically for the fertility clinics to review their current practices in order to resume the fertility services and share their own perspectives with other professionals.

## Data Availability

Not applicable

## Abbreviations

ACE2: Angiotensin converting enzyme 2
ART: Assisted reproductive technology
COVID-19: Coronavirus disease 19
HBV: Hepatitis B virus
HCV: Hepatitis C virus
MERS-CoV: Middle East respiratory disease coronavirus
RNA: Ribonucleic acid
RT-PCR: Reverse transcriptase polymerase chain reaction
SARS-CoV-2: Severe acute respiratory syndrome coronavirus 2
TMPRSS2: Transmembrane protease, serine 2
ZIKV: Zika virus
